# Research on Spatiotemporal Continuous Information Perception of Overburden Compression–Tensile Strain Transition Zone during Mining and Integrated Safety Guarantee System

**DOI:** 10.3390/s24175856

**Published:** 2024-09-09

**Authors:** Gang Cheng, Ziyi Wang, Bin Shi, Tianlu Cai, Minfu Liang, Jinghong Wu, Qinliang You

**Affiliations:** 1 School of Computer Science, North China Institute of Science and Technology, Beijing 101601, China; chenggang@ncist.edu.cn (G.C.); wangziyi@ncist.edu.cn (Z.W.); youqinliang@ncist.edu.cn (Q.Y.); 2 School of Earth Sciences and Engineering, Nanjing University, Nanjing 210023, China; 3 Key Laboratory of Earth Fissures Geological Disaster, Ministry of Natural Resources, Nanjing 210018, China; 4 Engineering Research Center of Zero-Carbon and Negative-Carbon Technology in Depth of Mining Areas, China University of Mining and Technology, Ministry of Education, Xuzhou 221116, China; 5 School of Mines, China University of Mining and Technology, Xuzhou 221116, China; liangmf2014@cumt.edu.cn; 6 School of Civil Engineering, Suzhou University of Science and Technology, Suzhou 215011, China; wjh@usts.edu.cn

**Keywords:** spatiotemporal continuous, overburden deformation, compression–tension strain transition zone, fiber optic neural sensing, integrated safety guarantee system, mining subsidence

## Abstract

The mining of deep underground coal seams induces the movement, failure, and collapse of the overlying rock–soil body, and the development of this damaging effect on the surface causes ground fissures and ground subsidence on the surface. To ensure safety throughout the life cycle of the mine, fully distributed, real-time, and continuous sensing and early warning is essential. However, due to mining being a dynamic process with time and space, the overburden movement and collapse induced by mining activities often have a time lag effect. Therefore, how to find a new way to resolve the issue of the existing discontinuous monitoring technology of overburden deformation, obtain the spatiotemporal continuous information of the overlying strata above the coal seam in real time and accurately, and clarify the whole process of deformation in the compression–tensile strain transition zone of overburden has become a key breakthrough in the investigation of overburden deformation mechanism and mining subsidence. On this basis, firstly, the advantages and disadvantages of in situ observation technology of mine rock–soil body were compared and analyzed from the five levels of survey, remote sensing, testing, exploration, and monitoring, and a deformation and failure perception technology based on spatiotemporal continuity was proposed. Secondly, the evolution characteristics and deformation failure mechanism of the compression–tensile strain transition zone of overburden were summarized from three aspects: the typical mode of deformation and collapse of overlying rock–soil body, the key controlling factors of deformation and failure in the overburden compression–tensile strain transition zone, and the stability evaluation of overburden based on reliability theory. Finally, the spatiotemporal continuous perception technology of overburden deformation based on DFOS is introduced in detail, and an integrated coal seam mining overburden safety guarantee system is proposed. The results of the research can provide an important evaluation basis for the design of mining intensity, emergency decisions, and disposal of risks, and they can also give important guidance for the assessment of ground geological and ecological restoration and management caused by underground coal mining.

## 1. Introduction

**Figure 1:**
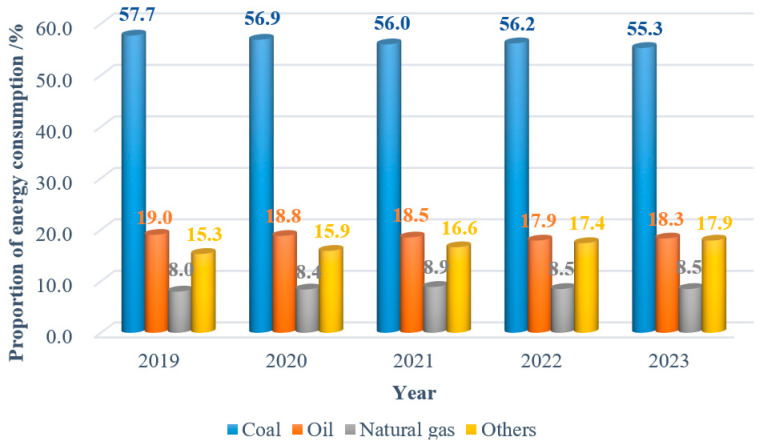
Structure of energy consumption in China, 2019–2023.

Over the past four decades, coal, as China’s basic energy, has provided a settled energy security for the accelerated growth of the national economy and the realization of national strategies. Strategic research conducted by the Chinese Academy of Engineering indicates that China’s coal-dominated energy structure will be difficult to change by 2050 [1]. The structure of energy consumption in China from 2019 to 2023 is shown in Figure 1 [2].

**Figure 2:**
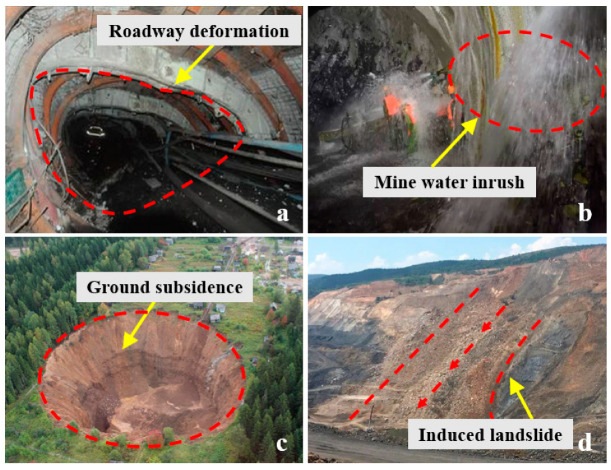
Mine accidents and geological disasters caused by mining: (**a**) Roadway deformation, (**b**) Mine water inrush, (**c**) Ground subsidence, and (**d**) Induced landslide.

The specific characteristics of China’s coal resources have a direct impact on the prevalence of shaft mining, which accounts for over 90% of the country’s total coal production [3]. As mining depth and intensity increase, particularly in mines with complex geological conditions, inadequate mining process and monitoring method design can result in disasters such as roadway deformation, mine water inrush, ground subsidence, induced landslides, etc. (Figure 2).

These disasters often result in significant casualties and property losses. For instance, on 22 February 2023, a significant coal mine collapse incident occurred in Inner Mongolia, China, leading to 53 fatalities and 204.3 million Chinese Yuan in economic losses. Similarly, on 25 February 2022, a major mine roof collapse incident took place in Guizhou Province, China, leading to 14 fatalities and economic losses of a further 22.9 million Chinese Yuan. On 10 April 2021, when a major mine water inrush accident occurred in Xinjiang Province, China, the incident resulted in 21 fatalities and 70.7 million Chinese Yuan in economic losses.

**Figure 3:**
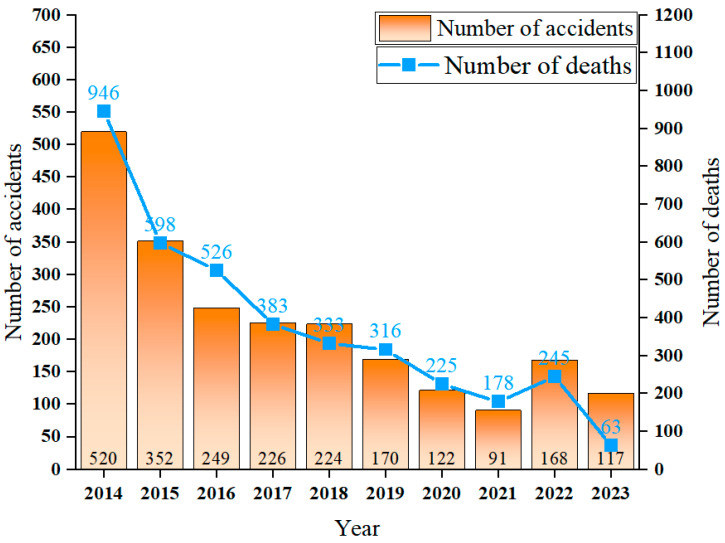
Statistics of coal mine accidents in China, 2014–2023.

In recent years, China has placed a significant emphasis on the supervision of mine production safety. This has led to the widespread utilization of diverse information technology, intelligent technology, and equipment in mining and disaster prevention and control. It also has effectively reduced the incidence of various types of mine disaster accidents and the degree of disaster and has resulted in a significant decrease in the occurrence of coal mine accidents and the mortality rate. However, the relevant economic departments of the Chinese government have published statistics indicating that the number of deaths per million tons of coal mines in China in 2023 will be 0.094. This figure remains elevated in comparison with that observed in developed countries. The statistics of China’s coal mine accidents in 2014–2023 are shown in Figure 3. These accidents and disasters frequently result from the deformation, movement, and collapse of overburden above coal seams, which are subject to influence from mining activities. This is particularly associated with the evolution characteristics of the overlying rock compression–tensile strain transition zone [4,5,6], which induces a significant menace to the safe production of mines and the protection of the surrounding environment. Consequently, it is urgent to prevent and control coal mine disasters and to guarantee safety throughout the mining process.

In summary, the stress state of the compression–tensile strain transition zone of overburden still lacks spatiotemporal continuous sensing means to obtain. The potential fractures and key interfaces of the overburden are difficult to accurately depict, which is the main bottleneck restricting the research to make breakthroughs; the relationship between the stress distribution characteristics of various strata, interfaces, and the overburden compression–tensile strain transition zone in the mining process has always been relatively unclear, which greatly hinders the mastery of the evolution characteristics and deformation failure mechanism of mining overburden. The understanding of the relationship between the characteristics of stress distribution within strata and between interfaces and the compression–tensile strain transition zone of overlying strata during mining has been relatively vague, and therefore, it greatly hinders the mastery of the evolution features and the collapse mechanism of overburden under coal mining activity.

As coal mining is a dynamic process with time and space, the deformation failure caused by the overburden often has a time lag effect, so it is necessary to adopt the overburden deformation sensing technology based on spatial and temporal continuity, accurately capture the deformation characteristic information of the overburden compression–tensile strain transition zone and its correlation criterion for stability evolution from the view of the interaction of overburden during coal mine based on the deformation failure control factors, to achieve the comprehensive cognizance from the deformation and movement in the overburden compression–tensile strain transition zone to the evolution mechanism of ground subsidence. It realizes the comprehensive knowledge of evolutionary mechanisms from the deformation of the overburden compression–tensile strain transition zone to the occurrence and development of ground subsidence. In view of this, the development and evolution of stope theory are systematically reviewed in this paper, the advantages and disadvantages of in situ observation technology of mine rock–soil body are compared and analyzed, and the evolution characteristics of the overburden compression–tensile strain transition zone and its deformation failure mechanism research are systematically summarized, the mining overburden deformation spatiotemporal continuous information perception technology based on distributed fiber optic sensing (DFOS) is introduced in detail. On this basis, an integrated safety guarantee system based on a fiber optic sensing neural network is proposed, which can offer a reference point for future mine safety monitoring research and practice.

## 2. Development of Overburden Deformation Theory and Its Mechanism Research

### 2.1. Evolution and Development of the Theory of Overburden in Mining

**Figure 4:**
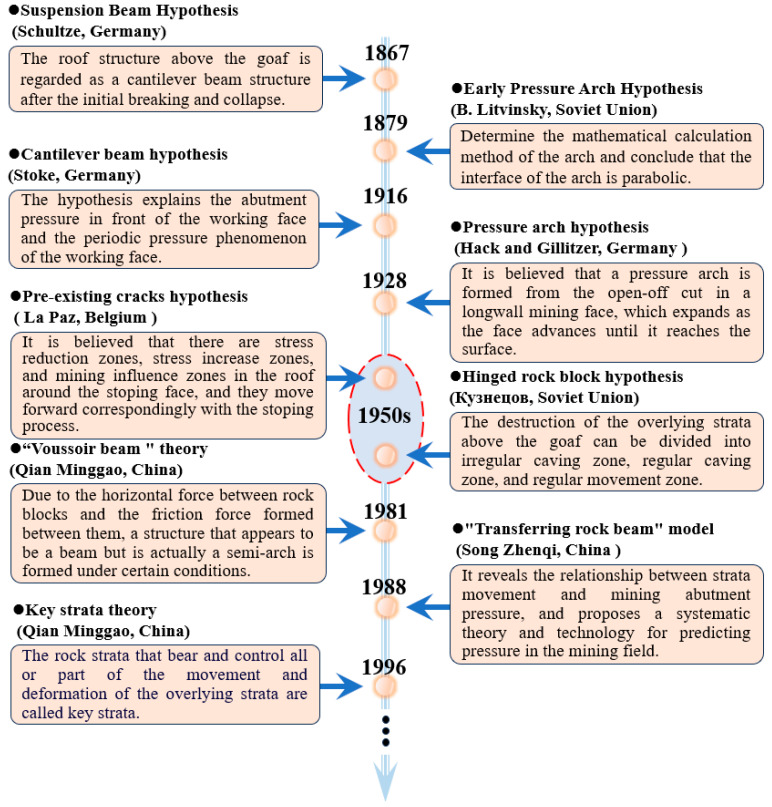
Development and evolution of stope structure model.

Overburden deformation is a classic topic of coal mining, for more than one hundred years, global scholars have around the topic to carry out a lot of theoretical research, continuously exploring the mining theory and observation methods that coordinate mining with the environment and have achieved some important results [7,8,9,10,11], numerous coal mining evolutionary theories have been developed, it provides an important reference for scientifically revealing the deformation, movement and collapse laws of the overlying rocks and the evolution process of the separation layer. The evolution of the development of the structural model of the stope is shown in Figure 4.

Overburden deformation failure is the major control element influencing mining security, in which the research on the overburden compression–tensile strain transition zone spatiotemporal continuous information perception and deformation failure mechanism is the key. During coal mining, the overlying rock strata are divided into rock groups that alternately appear as bending–separation–closure, with the workface continuing to advance, the overlying rock in the mining area occurs periodic separation–fracture–collapse, the overburden deformation has obvious zoning characteristics, there is a concentration of lag phenomenon, and the strata subsidence movement shows a periodic change trend with the advance of the workface.

**Figure 5:**
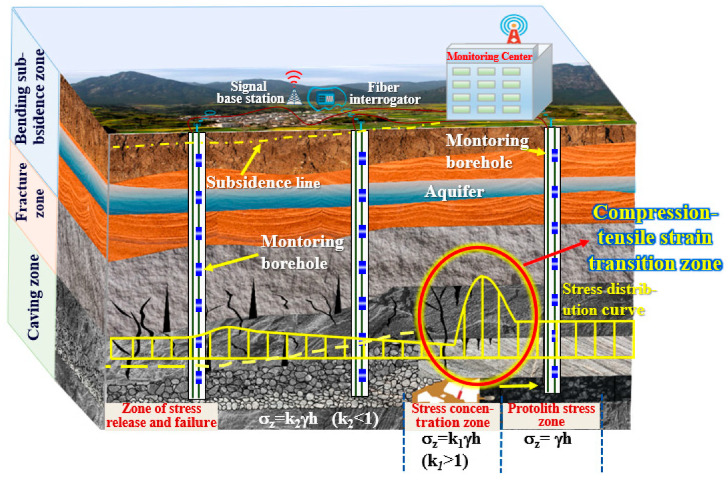
Theoretical model of overburden stress distribution. (Where: σ_z_ is the peak stress of coal pillars; *k* is the stress concentration coefficient; γ is the average bulk density of the overlying rock layer of the coal seam, k/Nm^3^; h is buried in the coal seam deep, m.)

In the above process, the overburden deformation experienced continuous and discontinuous compression and tension processes, forming multiple overburden compression–tensile strain transition zones, the distribution characteristics of these transition zones, their deformation and failure processes often control the scope of deformation and failure to the overlying strata above the coal seam as well as the extent of the disaster; simultaneously, under the influence of various geological situations and mining process parameters, the distribution range, extent and evolution characteristics of the compression–tensile strain transition zone of the overburden are all different, so it is crucial to clarify the transition process of the overburden from compressive strain to tensile strain for analyzing the overburden stability and failure mechanism. Figure 5 shows the theoretical model of stress distribution.

### 2.2. Research on In Situ Observation Technology for Overburden Deformation

Owing to the diversities in the occurrence and evolution of deformation in the compression–tensile strain transition zone of overlying rock strata under different geological conditions, the deformation and failure in the overburden compression–tensile strain transition zone is an evolving process, the longitudinal and transverse deformation of the overburden and the coal seam will alter dynamically. Accordingly, the deformation development trend and interaction between the overlying rock strata above the coal seam have a strong space–time effect, and the majority of the current studies do not take into account this spatiotemporal continuous processes, so they have difficulty in fully revealing the disaster principles of the overburden compression–tensile strain transition zone. Due to the imbalance between the level of mining monitoring technology and the high-level development of the mine, there are still many challenges in effectively obtaining the distribution location and development characteristics of the overburden compression–tensile strain transition zone. In the process of the above theoretical evolution, scientific researchers in the field of mining have carried out a series of studies on the deformation and failure process of overburden and separation evolution law, formed a variety of monitoring methods, such as engineering geological analogy, simulation of similar physical model experiments, numerical computation and field tests, etc. However, because of the large differences in geological conditions of mining areas, and the influence of different mining methods and technologies on coal seams, it is difficult to carry out effective research by using traditional theoretical methods; the analysis of overburden failure law must be combined with long-term and reliable observation data for comparison and correction; the selection of parameters in numerical computation is characterized by a high degree of randomness, and the results obtained from the different software exhibit some degree of difference, as well as it is difficult to simulate the mining of coal seams with complex geological structures by model tests. The reason for this is that the data obtained by such methods and techniques are discrete and intermittent in time and space. Therefore, in the monitoring of coal seam mining, there is an urgent need to strengthen the in-situ observation technology of rock–soil body technology innovation, especially the spatiotemporal continuous sensing technology, to acquire more holistic and dependable field test data, and based on which to carry out the prediction of periodic cumulative data and the study of mutation data, so as to realize the sensing of spatiotemporal continuous data of overburden deformation and the accurate calculation of ground subsidence value, as well as the evaluation of subsidence development potential. To clarify the advantages, disadvantages, and applicability of various in situ observation techniques, the in situ observation technology of the overlying rock–soil body is summarized systematically in this paper, which is shown in Table 1.

The aforementioned methods provide a crucial reference for the research on overburden deformation and failure mechanism; however, in terms of the spatial and temporal continuity of deformation data, the current observation techniques of overburden deformation can be roughly divided into five types of survey, remote sensing, testing, exploration and monitoring, and there are the following limitations pertain to the application of this approach to the study of deformation and failure law of overburden rocks: (1) the conventional survey techniques are discontinuous in time and space, and dependent on the experience of the surveyors; (2) the testing and exploration techniques are discontinuous in time, and cannot to obtain the real-time state information of the overlying rock–soil body; (3) the majority of monitoring technologies are point-type, dispersed data and discontinuous in space, and are unable to comprehensively capture the accumulated deformation of the overlying rock–soil body caused by continuous mining. In the current mine safety monitoring, it is difficult to accurately obtain the spatiotemporal continuous information of overburden deformation, and then it is difficult to capture the abnormal sign information before disaster through the distribution location and evolution characteristics of overburden compression–tensile strain transition zone and send out the early warning signals in time so that the evacuation of personnel can have enough time to avoid the risk of danger; at the same time, it is even more difficult to predict, prevent and control the geological disasters and ecological environmental damage on the ground surface due to the time lag effect. Therefore, there is an urgent need to grasp the whole process of deformation and failure of geotechnical bodies through the instantaneous perception technology of spatiotemporal continuous information, so as to ensure the accurate research and assessment of all kinds of mine disaster risks and scientific disposal, and in order to have a provision of critical technical support for the realization of the intrinsic safety of mine production and green development of mining areas. At present, the main means to realize the deformation of mine rock cover space–time continuous information perception is the DFOS technology.

### 2.3. Researcher on the Evolution Characteristics and Deformation Mechanism of the Compression–Tensile Strain Transition Zone

The final target of overburden deformation monitoring is to achieve safe mining and disaster prevention and mitigation of mining subsidence, however, in practice, as the mining action is a dynamic process with time and space, the deformation induced by coal mining is often time-lagged, so to take full account of the geological conditions of the area to be monitored, and on the basis of considering the deformation failure control factors, viewed from the interaction between the various rock strata overlying the coal seam. It is necessary to accurately capture the correlation criterion between overburden deformation characteristic information and its stability evolution, and then realize a full comprehension of the evolutionary mechanism from movement and failure of overlying rock strata to the occurrence and development of ground subsidence, which is the fundamental of realizing the assessment of the stabilization of overburden above the coal seam.

#### 2.3.1. Typical Modes of Overburden Deformation

**Figure 6:**
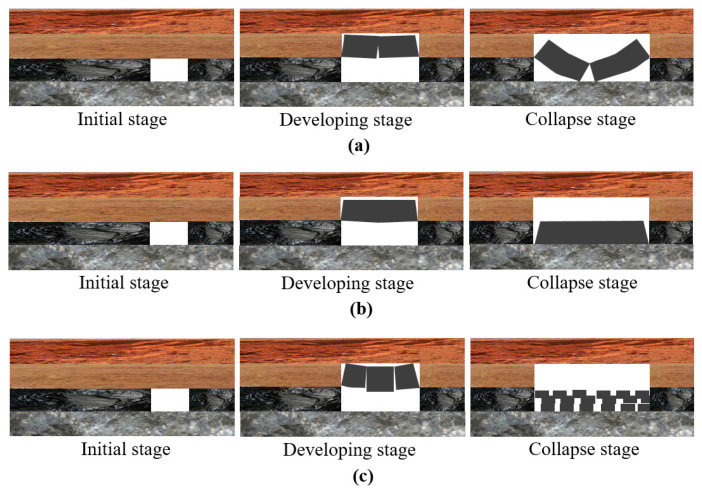
Typical types of overburden deformation: (**a**) Bending and tensile failure, (**b**) Overall shear failure, and (**c**) Shear and sliding failure.

Because the coal seam has experienced a long period of sedimentation, a large number of random mechanical discontinuity interfaces are generated in the overburden, which is often the key to the disaster of the geotechnical body, and the overburden deformation has a strong dynamic, especially the deformation of the rock–soil body at the depth of ground subsidence development, which has an intense temporal effect. Therefore, in practice, it is necessary to collect the relevant measured data of the overburden deformation under typical geological situations, and based on the geological conditions such as stratum distribution, coal seam thickness, lithologic parameters, and groundwater occurrence in the study area, combined with the historical mining parameters, the disaster characteristic information that can reflect the deformation of each stratum above the coal seam is excavated [12,13,14,15,16,17] and combined with the typical modes of overburden deformation: (1) bending and tensile failure, (2) overall shear failure, (3) shear and sliding failure (Figure 6). The coal-mining-induced failure mode of overburden is analyzed, which gives a reference basis for the investigation of the deformation mechanism of overburden under different geological conditions and realizes the scientific disaster prevention and mitigation of coal seam mining disaster and ground subsidence, as well as the green development of geological environment restoration.

#### 2.3.2. Key Controlling Factors of Deformation in the Compression–Tensile Strain Transition Zone of Overburden

**Figure 7:**
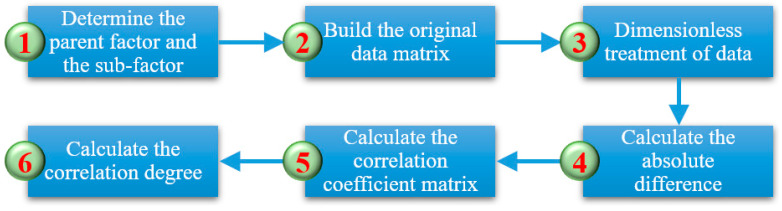
The process of gray relational analysis.

The deformation and movement of overlying rock–soil bodies cause ground subsidence, and this process is affected by many factors to clarify the key control factors of deformation. Based on the relevant theory of mining subsidence, nine factors that are highly correlated with deformation and failure in the compression–tensile strain transition zone of the overburden and ground subsidence were preliminarily selected, combining the results of model tests and numerical simulations from two major aspects, geological conditions and mining technology, supplemented with consideration of additional factors (Table 2), and then, gray relational analysis was used to analyze the sensitivity of each factor and quantify the influence of each factor by orthogonal test to determine the decisive factors in the system. The process of gray relational analysis is shown in Figure 7.

**Figure 8:**
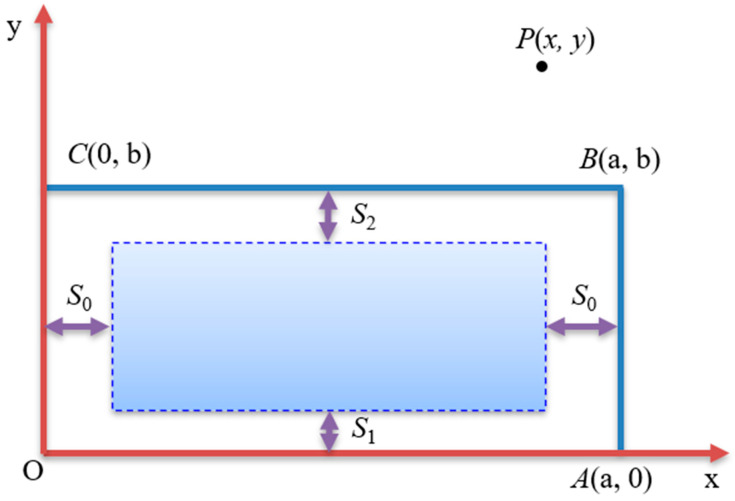
Schematic diagram of probability integration method.

The traditional probabilistic integral prediction model can predict the regular mining area (Figure 8), and the OABC is a mining face, assuming that none of the areas are mined outside the area, and the offset distances of the strike, downhill, and uphill inflection points are *S*_0_, *S*_1_, and *S*_2_, respectively.

The following formula is used to calculate the deformation of movement at any point on a surface by the integral method of probability:

(1) $$W\left(x,y\right)=f\left(t\right)W_{max}\iint_{D}\frac{1}{r^{2}}e^{-\pi \frac{{\left(\mu -x\right)}^{2}{+(\lambda -y)}^{2}}{r^{2}}}d\mu d\lambda$$

where $W_{max}$ is the maximum subsidence value of fully mined surface, *D* is the mining area, *r* is the mining radius, x,y are to calculate the relative coordinates of points (consider inflection point offset distance).

Then, we can know that the sinking amount of *P* (*x*, *y*) is

(2) $$W_{P}\left(x,y\right)={\;W}_{max}\int_{\mu ={\;S}_{0}}^{\tau =B-S_{2}}d\mu \int_{\tau ={\;S}_{0}}^{\tau =a-S_{0}}\frac{1}{r^{2}}e^{-\pi \frac{{\left(x-\tau \right)}^{2}{+\left(y-\mu \right)}^{2}}{r}}d\tau =W_{max\sum_{1}^{n}\Delta W(\tau ,\mu )}$$

where $W_{P}\left(x,y\right)$ is the sinking amount of any point $P\left(x,y\right)$, and the corresponding parameters are shown in Equation (1).

**Figure 9:**
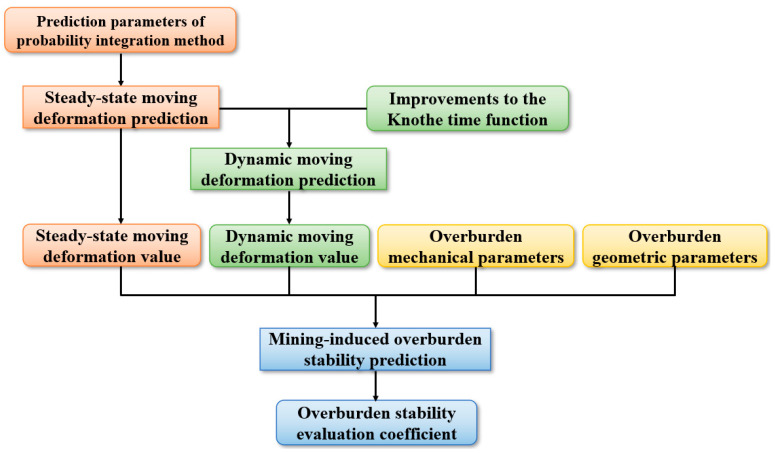
Prediction process of overburden stability.

Based on the above method, the subsidence value in the idealized state can be obtained, and the improved probability integration method is used to obtain the prediction of the mining subsidence value that conforms to reality. Based on this, considering the nonlinear mechanical properties of the overlying rock strata, the nonlinear variation characteristics of failure of the rock strata with time after coal mining, combined with the spatiotemporal continuous overburden deformation perception data, the improved Knothe time function is introduced, and the stability evaluation coefficient of the overburden is obtained (Figure 9), which is compared and corrected according to the field measurement data. The accurate identification of the deformation control factors and the quantitative evaluation of their influencing effects in the compression–tensile strain transition zone of overburden can be realized, which offers a reference for the stability assessment of overburden.

#### 2.3.3. Research on Stability Evaluation of Mining Overburden Based on Reliability Theory

**Figure 10:**
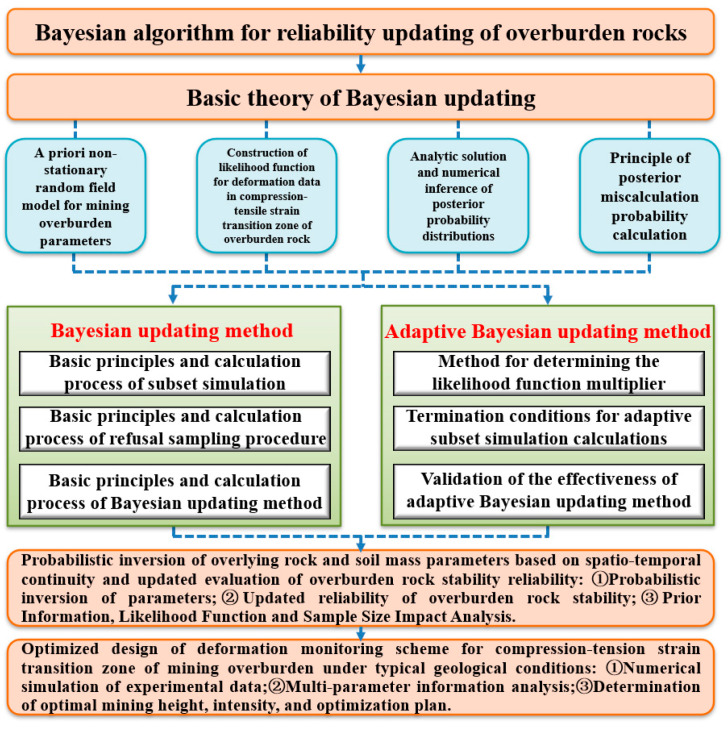
Bayesian-based overburden rock stability evaluation.

According to the multivariate Bayesian reliability theory, by fusing the prior information of the overlying rock–soil body mining parameters (lithological structure, geological structure, in situ stress, etc.) and external influencing factors (mining speed, strength and height, etc.), the probability distribution of rock and soil parameters is inferred by making full use of the testing data, monitoring data and multiparameter information, and the expected compression–tensile strain threshold is determined based on the adaptive strategy, and the probability inversion and reliability update evaluation of the overlying rock and soil parameters during mining are carried out (Figure 10).

The data mining algorithm was introduced to construct the overburden deformation prediction model, the control standards for overburden deformation and mining subsidence were formulated based on the reliability index, the decision function and Bayesian network classifier were used to identify the state and evolution process of the compression–tensile strain transition zone of the overlying rock–soil body, and the stability criterion system of the mining overburden was established from the perspectives of interface distribution, stratum displacement, “two-zone” (caving zone and fracture zone) height, separation development, geothermal gradient, etc., and the analysis results of the transformation mode, rock strata strength, and deformation features of the compression–tensile strain transition zone under mining were synthesized. Prediction of the location and time of the occurrence of abrupt change points is carried out, simultaneously considering the periodic deformation data and combining the stratigraphic section and field measurement data to enable the accurate assessment of the stability of overburden.

## 3. Research on the Spatiotemporal Continuous Information Sensing and Integrated Safety Guarantee System

### 3.1. Spatiotemporal Continuous Information Sensing for Overburden Deformation Based on DFOS

**Figure 11:**
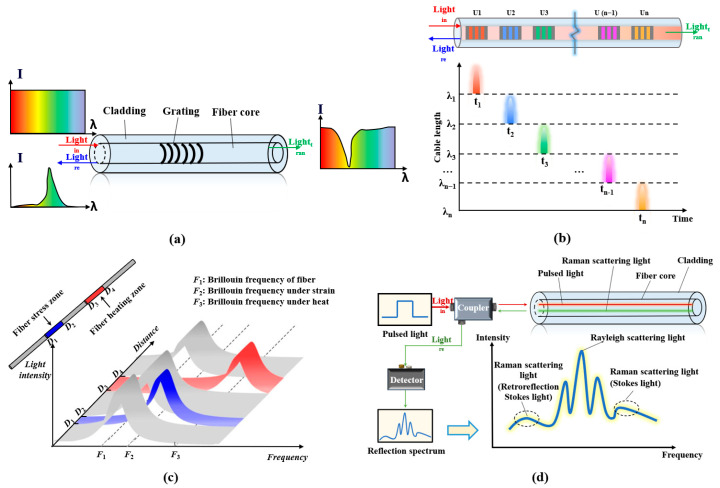
Principle of FBG and DFOS technologies: (**a**) FBG (Fiber Brag Grating), (**b**) UWFBG ((Ultra-Weak Fiber Bragg Grating), (**c**) BOTDR (Brillouin Optical Time Domain Reflectometry), and (**d**) DTS (Distributed Temperature Sensing).

DFOS is a novel sensing technology using light as a carrier and fiber as the transmission medium. This technology primarily employs optical fibers as sensors to perceive changes in environmental parameters through variations in the frequency, wavelength, phase, and other attributes of the optical signals within the fibers. Additionally, the optical fibers serve as the transmission medium for these signals, enabling the acquisition of vast amounts of sensing information along the monitored fiber route through a single fiber. Based on modulation and demodulation methods, fiber optic sensing technology can be further subdivided into several categories, including fiber Bragg gratings, Brillouin scattering, and Rayleigh scattering (Figure 11) [18].

The comparative analysis of various fiber optic sensing technologies is summarized in Table 3. Since strain, temperature, and vibration are the primary parameters for monitoring and early warning of geological disasters and engineering projects, three significant branches have emerged, Distributed Strain Sensing (DSS), Distributed Temperature Sensing (DTS), and Distributed Acoustic Sensing (DAS), collectively known as the “3Ds” [19]. This type of technology not only possesses advantages such as electromagnetic interference resistance, small size, high sensitivity, and good durability but also enables real-time acquisition of physical quantities such as strain, temperature, and vibration at various positions along the optical fibers. By constructing a multiparameter sensing network according to specific requirements, it is akin to implanting perceivable “nerves” into the earth, constantly capturing omen information of geological disasters in the rock–soil body.

**Figure 12:**
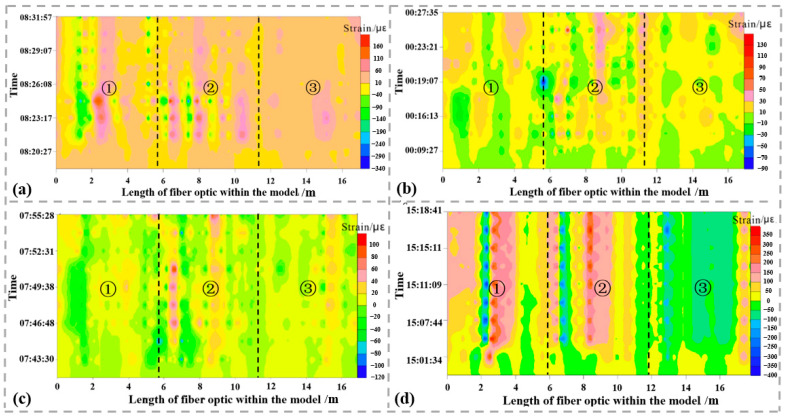
Temperature test results at different leakage pressures (**a**–**d**). Strain changes in sensing cables in different layers (leakage pressure of 1 MPa). ① represents the bottom layer; ② represents the middle layer; ③ represents the top layer.

Over the past decade, owing to the speedy development of mobile communication, fiber optic sensing technology has swiftly emerged as an innovative approach to geological engineering safety monitoring [20,21,22,23]. In particular, during on-site monitoring, fiber optics can be arranged in linear, ring, or mesh patterns based on the monitoring requirements of the target, enabling continuous monitoring of the target. As a result, it has been widely applied to the health monitoring of significant infrastructure projects, for instance, oil and gas pipelines, slope engineering, tunnel engineering, and mine engineering. Zhang et al. [24] employed DFOS technology based on Brillouin Optical Frequency Domain Analysis (BOFDA) to monitor the strain around pipelines. By installing strain sensors and actively heating fiber optics around the pipeline, combined with the point source leakage model, it can obtain information on gas pipeline leaks. The research results indicate that during a 1.0 MPa gas leak, the strain and temperature behavior of pipes varies within the range of −392–402 µε at different pressures. When the model is subjected to gas leakage pressures ranging from 0.3 to 1.0 MPa, marked changes in strain and temperature occur (Figure 12). Furthermore, the copper net of the proactively heated fiber optic is able to precisely identify the position of the leakage. The aforementioned findings of the study offer strong support for determining the leakage location of underground gas pipelines.

Judit Gómeza et al. [25] applied DFOS technology to monitor the lining of the Barcelona subway tunnel, which was influenced by nearby construction. They employed an algorithm to identify and replace outliers (outliers are mainly caused by the failure of fiber-reinforced concrete bonding technology, the existence of geometric discontinuities that need to be overcome, such as corners, joints, etc., or errors generated by the sensor itself) in the raw data set. By doing so, they obtained a smooth strain trend surface. The tunnel deformation process was accurately reproduced, justifying the dependability of the DFOS technology in data monitoring.

**Figure 13:**
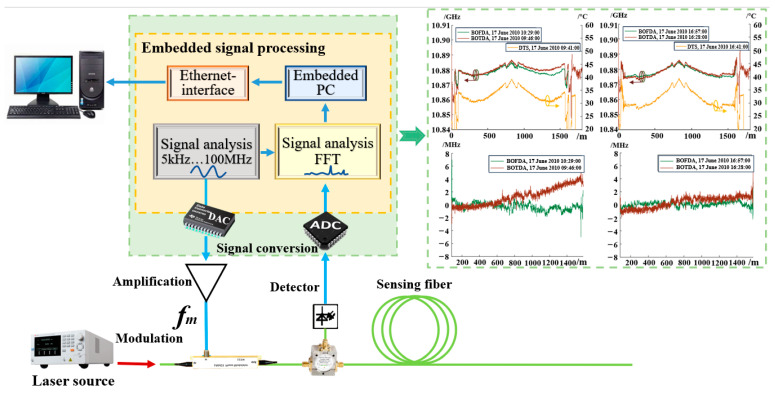
Set-up of digital BOFDA system (“DAC” represents Digital to Analog Converter; “ADC” represents Analog to Digital Converter).

In particular, with the significant improvement in the performance and deployment technology of fiber optic sensors in the last few years, the appliance of DFOS technology in mine engineering safety monitoring has become a hot topic in the global field of safe mining. N. Nöther et al. [26] installed fiber optic sensors on the outer wall of a shaft reaching a depth of 800 m, enabling uninterrupted monitoring for 5 days within the strain and temperature of the shaft, utilizing BOTDA measurements as a reference, the BOFDA technology was employed to monitor the strain in boreholes. The set-up of the digital BOFDA system is illustrated in Figure 13.

Additionally, distributed temperature measurements were conducted by using DTS. The study confirms the viability of using Brillouin and Raman sensing technologies for monitoring in the borehole. By applying temperature compensation to the Brillouin measurements through DTS measurements, the effect of temperature on the results can be eliminated, enabling more accurate deformation values of the object under test to be obtained.

**Figure 14:**
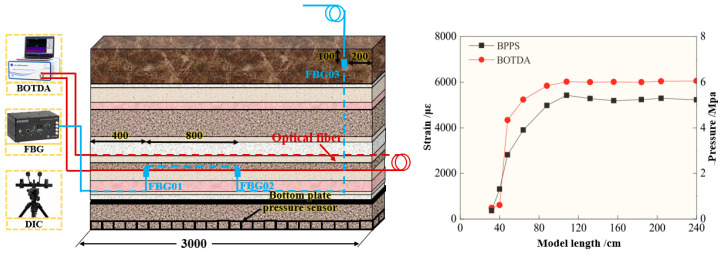
Monitoring system layout and result.

Chai et al. [27] employed BOTDA, FBG, and Digital Image Correlation (DIC) technologies to monitor the deformation of key strata in the overburden. DIC is an optical measurement experimental technology that combines digital image processing technology and modern optical mechanics, which obtains the displacement vector by tracking the position change in the same pixel before and after the deformation of the object and then obtains the full-field displacement and strain information of the monitored object to realize optical fiber monitoring. It has the characteristics of high precision, strong flexibility, strong anti-interference ability, and wide measurement range. By combining the fracture mechanics model of the key stratum and the function model of subsidence, a spatial stress evolution model for overburden failure under coal mining has been established, quantitatively characterizing the relationship between pressure changes in the goaf and the failure of key strata. The layout scheme of the test and results is shown in Figure 14.

**Figure 15:**
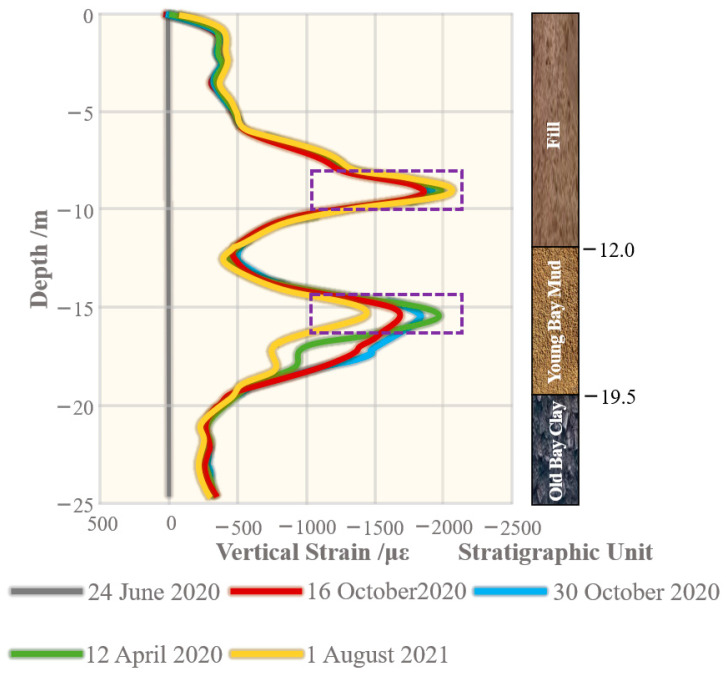
Strain curve of decoupling test.

Kenichi et al. [28] adopted DFOS technology to obtain continuous strain profiles of soil deformation under stress, thereby enabling continuous measurement of underground deformation. Furthermore, an installation technique for sensing cables was proposed, which involved direct pushing alongside Cone Penetration Testing (CPT), effectively correlating strain localization with soil resistance encountered during CPT. The research process revealed that while fiber decoupling occurred during measurements of higher strains, calculations performed on data measurements below 2000 µε demonstrated good consistency with actual measurements of surface settlement. The strain curve from the fiber optic test is depicted in Figure 15. According to the data curve from 8 January 2020, the decoupling of the sensing cable occurred within the Young Bay Mud layer. Therefore, for deformation monitoring of such rock–soil body, it ought to be ensured that the cable has good coupling with the surrounding geotechnical body by increasing the anchorage points or improving the backfill material and laying method according to a certain spacing on the cable.

**Figure 16:**
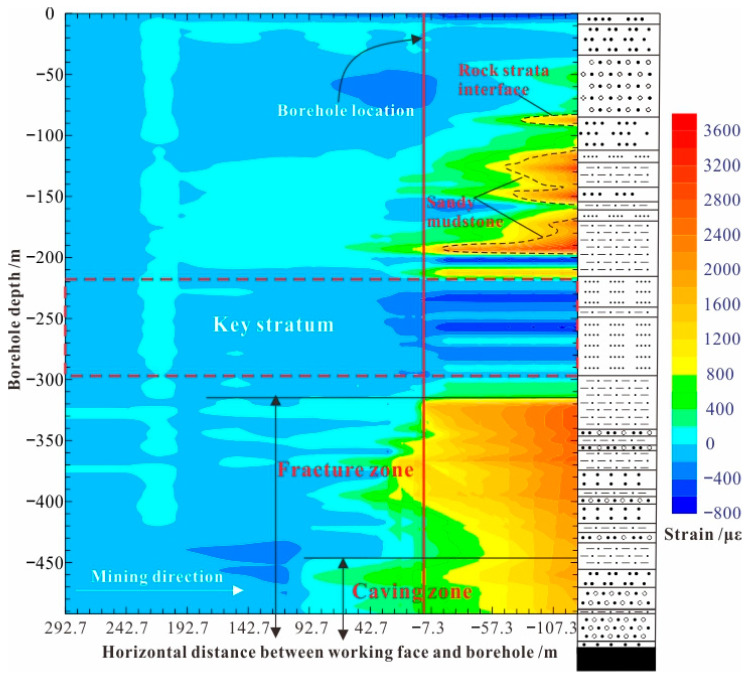
Strain distribution of overburden deformation.

Sun et al. [29] employed BOTDR technology and the resistivity method, analyzing the “optical–electrical” evolution law of overlying rock strata in the process of coal mining. The investigative results show that the two parameters that have a significant influence on the failure mode of the overlying rock strata are the lithology of the roof and the stratigraphic structure; the rock strata with more soft or cracks are the first to undergo deformation and failure; the collapse and stress state of the rock strata develops in a “step shape” manner from the bottom to the top, and the stresses have the characteristics of zoning and temporal sequences in the horizontal and vertical directions; and the heights of the development of caving zone and fracture zone are about 75 m and 205 m, respectively, and the distance affected by shear stress in the early stage of coal mining is about 92.7 m (Figure 16).

The aforementioned research plays a crucial role in ensuring safe mining in mine engineering, controlling ground subsidence, and promoting green rehabilitation of goaf areas. However, the deformation course of the overburden rock–soil body above coal seams is often discontinuous, and as the mining depth and height of coal seams sustained growth, the value of overburden deformation caused by coal mining as well exhibits an upward trend, leading to a more frequent occurrence of large rock deformations. Taking China as an example, due to the diverse coal occurrence conditions, geological conditions, and mining methods across major coal-producing regions, there are certain discrepancies as it happens, development, and evolutionary process of deformation and failure of deep rock masses in mines. Furthermore, coal mining is a dynamic process that varies in time and space, and deformation and failure of the overlying rock–soil body caused by it often present a time lag effect. Therefore, there are high requirements for sensor selection and installation techniques, which must fully consider the geological conditions of the monitoring area. On the basis of considering the control factors of deformation and failure, sensors with a large measuring range, high durability, and continuous monitoring capabilities should be selected to conduct deformation monitoring of overlying rock strata. This approach enables precise capture of deformation characteristics in the compression–tensile strain transition zone, thereby achieving continuous spatiotemporal sensing of overburden deformation and the early warning and prediction of disasters. However, current deformation monitoring of mining-induced overlying strata above coal seams often relies on point-type sensors, which yield relatively discrete monitoring data and fail to achieve continuous spatiotemporal sensing of strata deformation. However, in the current monitoring of overburden deformation during coal mining, point-type sensors are often used, and the monitoring data obtained are scattered, which cannot realize spatiotemporal continuous sensing, and the sensors are less durable, and the system is not robust, so it has difficulty in tracking the cumulative deformation comprehensively and stably due to the continuous coal mining.

### 3.2. Research on the Coupling Performance between Sensing Cable and Rock–Soil Body

The core of the internal deformation of the formation using fiber optic sensing technology is the deformation of each stratum, especially the compression–tensile deformation of the rock strata interface, which can form a one-to-one correspondence with the deformation of the sensing cable itself, so the borehole backfill material, installation process, and coupling performance are the key to whether the results are effective and accurate; simultaneously, the robustness of the sensing system can be ensured [30,31,32].

#### 3.2.1. Backfill Material Selection

**Figure 17:**
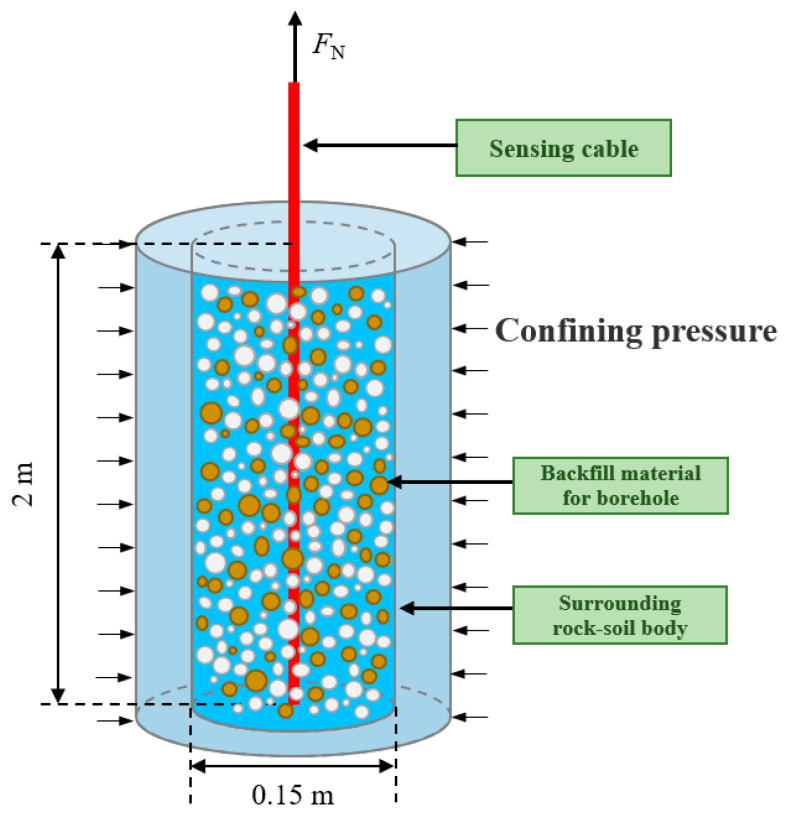
Backfilling material test model.

In the field test of deformation monitoring of mining overburden, the backfill materials used are usually classified into cement-based, chemical, and composite grouting materials. In the selection of backfill materials for boreholes, the viscosity, mechanical properties, impermeability, volatility, and other performance indicators of the grouting slurry must be considered in an integrated manner. Therefore, the on-site monitoring needs to be combined with the stratigraphic profile to backfill in sections for the following reasons: 

(1) in view of the large depth of the ground borehole for overburden deformation monitoring, its depth usually more than 500 m. For the geological conditions in the most mining area, most of them have reached the bedrock. Therefore, for the borehole in bedrock section, considering the grouting speed and cost, cement grout is usually used for grouting directly;

(2) for the fracture development layer section borehole, more permeable chemical grouting fluid or composite grouting fluid is used for grouting to better solidify the fracture zone and fracture development area; and (3) for the loose layer backfill, due to the strong compressibility of this section of the stratum, in practice, in situ soil mixed with an appropriate amount of bentonite is often used for backfill to ensure the coupling of the cable and the geotechnical body in the loose layer of the borehole. In addition, on-site monitoring is required in order to accurately calculate the amount of backfill material, which is often based on the length of the bedrock section of the borehole for calculation; at the same time, the process of grouting the top of the borehole should be used as an auxiliary part of the bundle drilling rod in order to avoid sensing cable damage due to grouting. The relevant experimental model is shown in Figure 17.

#### 3.2.2. The Installation Process of Sensing Cables

The deployment process and its quality have a marked influence on the monitoring results. Therefore, in the safety monitoring of mine engineering, the corresponding deployment methods should be selected according to different test environments and objects [33,34,35]. The sensing cable layout for overburden deformation monitoring usually adopts the method of direct burial, that is, all kinds of strain sensing cables and sensors are implanted into the rock–soil body through the ground borehole. Because the two processes of borehole construction and sensing cable implantation are independent of each other in the process of laying directly buried sensing cables, and because the construction efficiency is high and the sensing cables have a high success rate, they are widely used in the current overburden deformation and failure monitoring. In addition, the method can also integrate the ground monitoring borehole and underground workface monitoring borehole to form an integrated monitoring network and realize the full section deformation monitoring of the overlying rock–soil body under mining activity.

**Figure 18:**
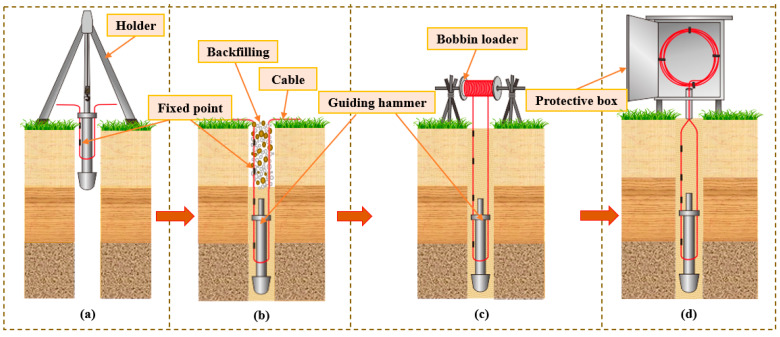
Sensing cable layout for the ground monitoring system: (**a**) Cable layout, (**b**) Borehole backfill, (**c**) Cable coupled with borehole, and (**d**) Cable protection.

The ground borehole laying method mainly focuses on the layout of the ground monitoring system. The primary task of the process is to select the monitoring area for the ground borehole, and then select the corresponding sensing cable (sensor) for laying according to the monitoring target and the geological situations of the monitoring area. The major steps are as follows: (1) Select a counterweight guide hammer. The guide hammer is selected according to the borehole depth, diameter, and the characteristics of the sensing cable (sensor) to guarantee that it guides the sensing cable (sensor) stably and efficiently. (2) Implant the sensing cable. Under the guidance of the counterweight guide hammer, the sensing cable is implanted into the borehole at a precise and uniform speed to ensure that it senses and transmits the information required for monitoring. (3) Borehole backfill and stabilization. The backfill material is selected according to the geological conditions to carry out segmented grouting of the borehole, and after the backfill is completed, the pigtail at the head of the sensing cable is welded after it is stabilized. (4) Sensing cable (sensor) protection. To guarantee long-term stable use of the sensing cable (sensor) in the project, it is necessary to take professional protection measures, such as installing protective sleeves and carrying out additional fixing treatment. The detailed steps of the sensing cable laying of the ground monitoring system are shown in Figure 18.

**Figure 19:**
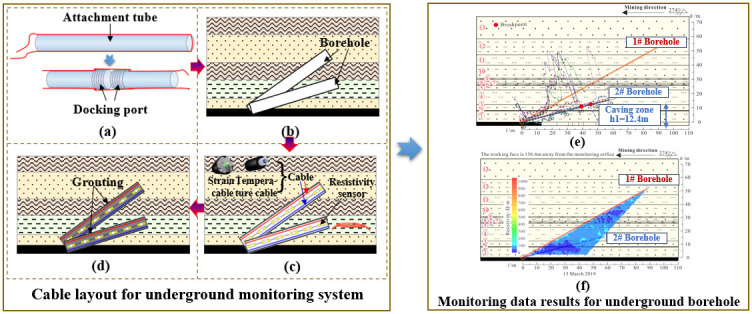
Sensing cable layout for the underground monitoring system: (**a**) Cable layout, (**b**) Borehole drill, (**c**) Grouting, (**d**) Cable implant, (**e**) Optic fiber monitoring result, and (**f**) Electrical method monitoring result.

The core of the underground laying method is to use lightweight pipe fittings with high strength as the attachment carrier of the sensing cable (sensor) and lay them in the overlying rock–soil body; the deformation of the rock–soil body can be effectively transmitted to the fiber core. Here are the main steps of the method: (1) By the practical situation of the coal mining workface, the borehole required for laying is accurately designed to make sure that the position and angle of the borehole satisfy the monitoring requirement. (2) The borehole in the required monitoring area is carried out in strict accordance with the designed plan to make sure that the borehole satisfies the demand. (3) The cable is attached to the lightweight pipe fitting, and it is fixed in the rock mass through grouting operation to form a stable monitoring network. (4) After the borehole achieves the expected coupling strength, collect the initial value of the cable and the obtained data, then conduct regular data collection based on the coal mining progress. The detailed process is shown in Figure 19.

#### 3.2.3. Borehole Coupling Experiments

**Figure 20:**
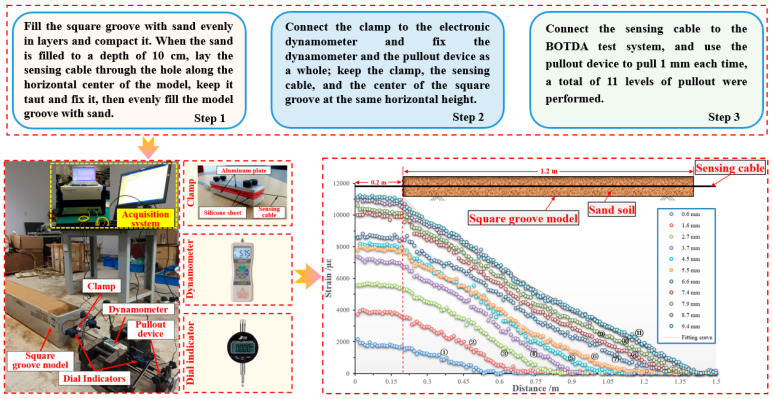
The layout process of pullout test and distribution of sensing cable strain data.

The coupling performance of sensing cable and rock–soil body is directly related to the effectiveness of monitoring data, which is the core problem of intelligent perception of overburden deformation during coal mining. Cheng et al. [36] adopted BOTDA technology to carry out a pullout test on the coupling performance, and the coupling performance between the sensing cable and sand was analyzed and judged. The transmission depth and strain distribution of the optical fiber during the pullout process are obtained, and a comparison, fitting, and evaluation of them with the theoretical calculation values are performed. Thus, the relationship between the deformation coupling performance of sensing cable and sand and factors such as the pullout displacement, the selection of backfill materials, and the type of sensing cable can be obtained. On this basis, the coupling evaluation indicators and standards can be established. The layout process of the pullout test and distribution of sensing cable strain data are shown in Figure 20.

Firstly, the initial value after sensing cable deployment is recorded. Subsequently, various displacement pullout operations are carried out, using a pullout device to pull out 1 mm each time, for a total of 11 levels of pullout, and the strain values of the optical fiber are measured after each pullout. Finally, the difference between the measured strain value and the initial value of the sensing cable can be calculated to obtain the strain distribution under various levels of pullout displacement.

The overall process is divided into three stages: (1) The fully coupled stage, known as the pure elastic phase. Due to the pullout force being relatively small at the beginning of the test, the interface between the sensing cable and the sand has good friction, making the coupling good at this stage. (2) The semicoupled stage includes three substages: elastic softening, pure softening, and softening residual. When the pullout force increases to a certain extent during this substage, the interface between the sensing cable and the sand begins to undergo shear failure and detachment, and the sand load on the sensing cable makes it difficult to support the shear failure force, resulting in displacement at the tail of the sensing cable, and the state between the sensing cable and the sand is semicoupled at this time. (3) Relative slippage stage, called the pure residual stage. At this stage, as the pullout force continues to increase, the shear layer between the sensing cable and the sand interface is gradually destroyed, causing relative slippage between the sensing cable and the sand interface. At this point, as the pullout force further increases, the sensing cable is gradually pulled out of the sand [37,38].

**Figure 21:**
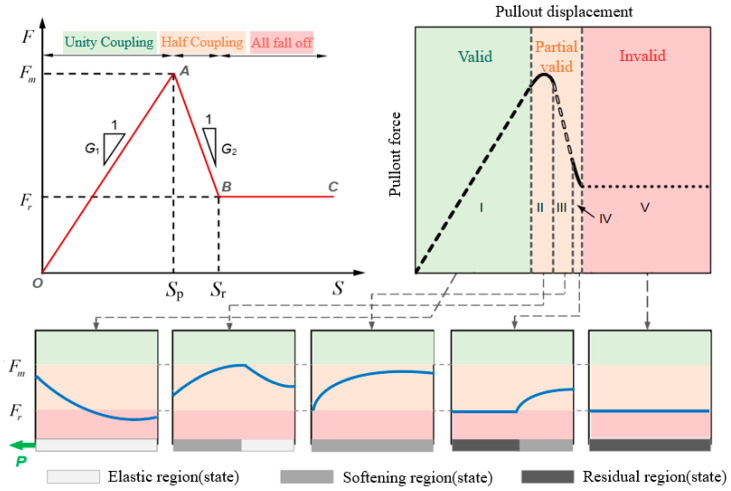
The three-stage model of pullout force–displacement relationship. The blue lines indicate different pull-out force distributions, and the five Roman numerals represent the five stages of pure elasticity, elasticity-softening, pure softening, softening-residual, and pure residual.

According to the action process of the interface between the cable and the sand in the above test, a pullout force–displacement relationship model is established, as shown in Figure 21.

**Figure 22:**
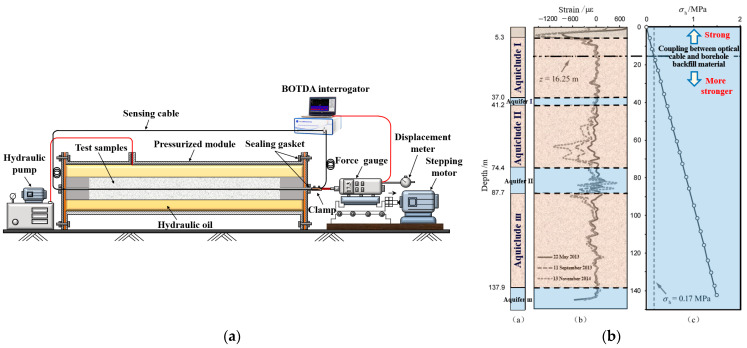
Coupling test for sensing cable–soil under controllable confining pressure: (**a**) diagram of test device; (**b**) curves of ground subsidence and calculated values of ground pressure [40].

Zhang et al. studied the coupling between sensing cables and loose-filled sand (compacted sand–clay soils) under different confining pressures [39]. By increasing the confining pressure control factors, the interaction mechanism between the sensing cable and the rock–soil body within the range of 0–1.6 MPa confining pressure was revealed; under a certain confining pressure, the sensing cables have strong coupling with the soil, and it was concluded that the cable has strong coupling with the soil under a certain confining pressure. Taking the land subsidence in Shengze, Suzhou, China, as an example, field measurements were conducted for verification to obtain the coupling coefficient ζc-s > 0.9 between the sensing cable and borehole backfill material below a distance of approximately 16 m from the ground surface. This indicates that the borehole backfill material has a strong coupling with the sensing cable, and the strain monitoring data of the sensing cable can accurately reflect the true deformation of the formation (Figure 22). For shallow formations, due to the relatively low confining pressure, on the one hand, the deformation monitoring data can be corrected based on the ζc-s value. On the other hand, anchoring points can be set at regular intervals along the length of the sensing cable to improve the coupling between the sensing cable and the borehole backfill material. In the monitoring of overburden deformation based on ground boreholes, the two methods mentioned above are accepted to improve low confining pressure in shallow formations. To ensure the coupling between the sensing cable and the rock–soil body is tested, in practice, it can be conducted according to the geological characteristics of the rock strata, selecting the undisturbed rock and soil corresponding to the formation to backfill the borehole.

In conclusion, currently, there is still a lack of advanced information technology to perceive the spatiotemporal continuity and refined deformation state of the overlying rock–soil body above coal seams under mining action, so the development characteristics and distribution law of overlying rock fractures are difficult to accurately characterize. The above issues are the main bottlenecks that restrict breakthrough progress in deformation and failure mechanisms investigation of overlying strata during coal mining. Therefore, based on fiber optic sensing neural networks, a security guarantee system that integrates perception, transmission, processing, warning, decision making, and emergency response can be established. On this basis, intelligent perception and real-time monitoring of the entire process of deformation and failure of overlying strata can be achieved. This fundamentally reveals the time-dependent disaster mechanism of overburden deformation and the spatiotemporal evolution law of mining subsidence, which improves the level of mining accident warning and forecasting and is of great significance.

### 3.3. Integrated Safety Guarantee System Based on Fiber Optic Perception Neural Network

**Figure 23:**
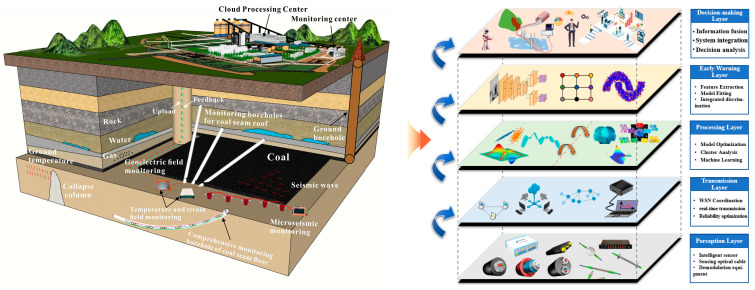
Integrated safety guarantee system for coal mining.

Due to the time lag of overlying rock strata deformation under mining activity, it is necessary to accurately capture the relevance between the representation of overburden deformation and their stability evolution based on the consideration of the geological conditions of the mine, as well as the controlling factors of deformation and failure. The above process presents certain limitations due to the current state of methods and technologies, which makes it challenging to obtain precise and real-time data regarding the deformation of the overlying rock–soil body, as well as the impact of multifield interactions on the deformation of roadways and ground subsidence. Consequently, an integrated safety guarantee system for coal mining is urgently being established (Figure 23) to break through the barriers of data perception, instant transmission, automatic processing, accurate warning, and scientific decision making interconnection. Furthermore, research must be conducted on the disaster mechanism of the entire process, from overlying rock strata movement to ground subsidence occurrence and evolution, with the aim of ensuring the intrinsic safety of coal mining and the sustainable restoration of ground subsidence.

#### 3.3.1. Perception Layer

**Figure 24:**
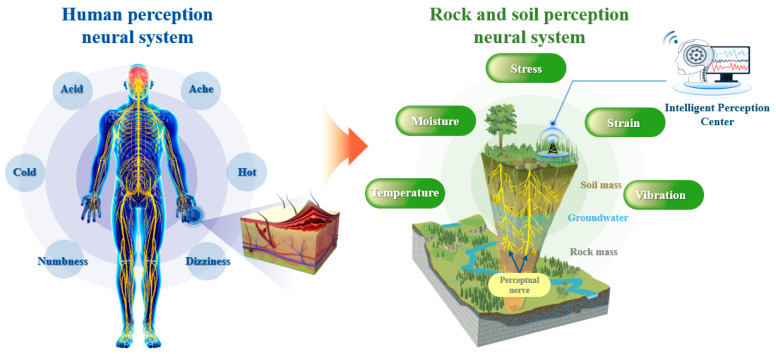
Neural perception of the rock–soil body.

The perception layer, located at the bottom of the whole system, is the core of the integrated safety guarantee system based on a fiber optic sensing neural network. Its primary function is to sense various physical parameters of the overburden above the coal seam under coal mining activity, including displacement, temperature, seepage pressure, resistivity, and vibration, as well as the environmental information within the monitoring area. This is achieved through the utilization of advanced sensing cables (sensors). Professor Shi, a Chinese scholar [41], based on his long-term understanding of the research and application of fiber optic sensing technology of geoengineering, compared with the human sensory nervous system, proposed the concept of the “neural perception” of geoengineering, that is, according to the monitoring target, by implanting longitudinal and transverse linear sensing elements in the rock–soil body to be measured, the geoengineering perception neural network was constructed to realize the “continuous time–space” perception of various parameters of the rock–soil body. For the human body, the perception layer is the five sense organs and the skin, which perceive the environmental state and its changes through sight, taste, smell, hearing, and touch; for the rock–soil body, the perception layer is the various sensing elements located on the surface and inside the rock–soil body. The dynamic identification of the state of geoengineering and its evolution process is carried out by various sensors of strain, temperature, moisture, and vibration (Figure 24).

The perception layer mainly consists of two parts: all kinds of mine monitoring intelligent sensors and demodulation equipment. The core of this layer is the integration of radio frequency, intelligent sensing, wireless networking, bus control, and other technologies to establish the connection between the rock–soil body being tested, the sensors, and the demodulation equipment. Among them, sensors are the basic unit to obtain the parameters of the rock–soil body. It converts all kinds of physical (chemical) parameter information and its changes in the rock–soil body into the changes in the photoelectric signals of sensors and then sends the signals to the demodulation equipment for preprocessing, calibration, and visual display. In coal seam safety mining monitoring based on fiber optic sensing, FBG, UWFBG, BOTDR, and other technologies are usually integrated, and the sensing cable (device) is deployed by using a ground borehole and underground workface borehole, so as to obtain the spatiotemporal continuous multiphysical field parameters and their changes in the overburden during coal mining in real time [42,43].

#### 3.3.2. Transmission Layer

The data information obtained from various sensors in the perception layer is packaged and sent to the transmission layer via short-range transmission technologies, such as demodulation equipment and system Fieldbus. The key technologies in this layer are mainly divided into wired transmission and short-range wireless communication. Wired transmission mainly uses optical communication cables to connect the sensing cables, sensors, and monitoring system, as well as transmit data to the processing layer after aggregation. Short-range wireless communication uses the sensor network to communicate with the perception layer. The sensor network is a transmission network consisting of sensor nodes. Each sensor node usually consists of sensing elements, microprocessors, and communication units. Each node works together through the communication network to complete the wireless transmission of multiparameter information about the rock, soil, and environment being measured. Due to the original data obtained from overburden safety monitoring being of various types and large amounts, it is essential that the information collected can be accurately transmitted to the processing layer, which places higher demands on the precision and dependability of perceptual data transmission. To address the reliability issues associated with the transmission of extensive data sets related to overburden deformation, a joint data acquisition scheme for ground and underground overburden monitoring has been developed. This scheme employs monitoring data demodulation technology, which relies on the Internet of Things (IoT) and wireless real-time transmission technologies, such as 5G, Wi-Fi, and Bluetooth. In practice, various portable high-performance measuring instruments and communication devices can be employed to establish an organic connection between ground vertical monitoring boreholes and underground workface upward inclined boreholes, thereby facilitating convenient data acquisition and transmission, which not only makes the monitoring range of mining-induced overburden failure wider, deeper, and more efficient but also realizes the development of monitoring content towards multisource, precision, and three-dimensionality.

**Figure 25:**
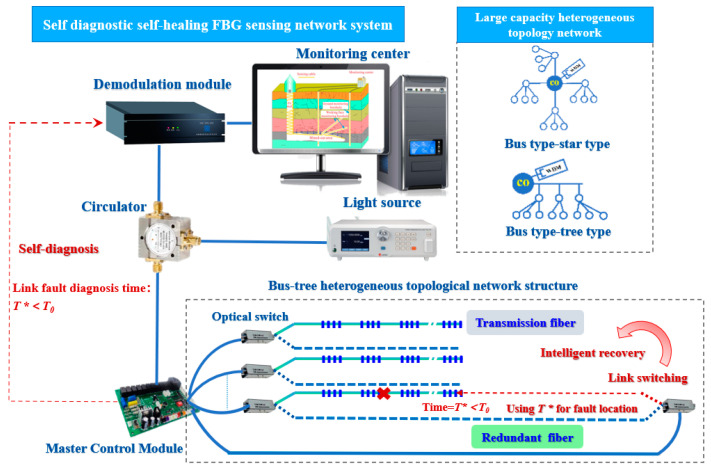
Self-diagnostic self-healing FBG sensing network system. The optical fiber has the ability of self-healing and self-diagnosis, and the cross sign indicates that after the upper fiber is broken, it can be switched to the following fiber for monitoring, so as to achieve uninterrupted monitoring. The dash lines indicate that the two fibers can be switched.

Most mine projects are located in relatively remote areas, and the monitoring points for mining-induced failures are scattered, making it difficult to repair faults in the monitoring network in a timely manner, and the system lacks self-healing capabilities. Therefore, a large-capacity heterogeneous fiber optic sensor network based on wavelength division, time division, and space division can be constructed to realize the synchronous monitoring of FBG multiparameter sensor array; various control schemes are used to equip the FBG sensor network with self-diagnosis performance. Employing redundant sensing fiber switching and other technical means, and according to the actual situation of optical path energy distribution, fiber sensor shape, optical link design, and demodulation system output, the optimal sensor network node control mode is selected to start the automatic recovery and adjustment mechanism of faulty sensors or transmission cables and build a large-capacity and high-robustness heterogeneous fiber sensor self-healing network to avoid the interruption of signal transmission at the fault point. Figure 25 shows the self-diagnosis and self-healing system of a large-capacity and high-robustness fiber optic sensor network [44].

#### 3.3.3. Processing Layer

During coal mining, the movement and collapse process of the overlying rock–soil body is extremely complex, and sudden collapse and shear fracture of the rock mass often occur, which makes the fiber optic sensing data disorder and abnormality. To this end, it is necessary to denoise and filter the abnormal fiber optic data. Wavelet transform, as a unique signal time–frequency analysis method, can perform multiresolution analysis; it can not only capture the subtle changes in the signal in the time domain but also show the local characteristics of the signal in the frequency domain. Cheng et al. selected the Daubechies wavelet to denoise the overburden deformation measurement data, effectively eliminating the effect of noise on data identification and analysis. A noisy signal model can be expressed in the following form:

(3) $$S\left(t\right)=f\left(t\right)+\sigma \cdot e\left(t\right),\;t=0,\;1,\ldots ,\;n-1$$

where *f(t)* is the true signal; *e(t)* is noise; and *S(t)* is the original signal. The objective of signal *S(t)* denoising is to remove the noise component of the signal, thereby facilitating the recovery of the underlying, unadulterated signal *f(t)*. The specific steps are as follows: (1) Implement wavelet decomposition of the original signal data. The db5 wavelet is employed to decompose the signal *S(t)* into four distinct layers. (2) Denoising threshold quantization. For the high-frequency coefficient of each layer, the project selects the principle of “unbiased risk estimation threshold” for threshold analysis and calculation. (3) Signal reconstruction. According to the low-frequency and high-frequency coefficients obtained by wavelet decomposition, the wavelet signal of the one-dimensional signal is reconstructed, and then the real signal FI is recovered.

In the on-site monitoring of overlying rock movement and collapse, with continuous coal mining, the sensing data obtained by various sensors in the perception layer are continuously accumulated. There is the question of how to classify, extract, modify, cluster, and optimize various sensing data quickly and accurately, realize the self-diagnosis and intelligent analysis of massive sensing data, and provide reliable and effective data sources for the subsequent development of mining-induced overburden deformation prediction modeling based on sensing data, which is a core function of the processing layer of this integrated system. In terms of intelligent processing of fiber optic sensing data, statistics, artificial intelligence (AI) big models, and filtering algorithms are used to realize intelligent denoising, interpolation, and anomaly identification of sensing data, as well as to improve the self-diagnostic level of data source reliability and the accuracy of intelligent analysis. Concurrently, a data mining methodology founded upon a topic model is employed to facilitate the intelligent processing of voluminous data. The principal procedure of this method is to utilize a neural network-based topic model. By introducing smooth prior and weak smooth prior into the topic model, the focus of text burst topic data is realized to effectively solve the sparsity problem of short-text data [45,46,47].

**Figure 26:**
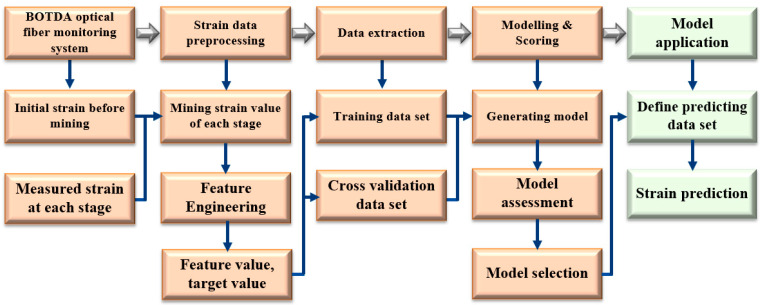
Modeling of overburden deformation prediction based on machine learning.

Liu et al. [48] integrated DFOS and machine learning technologies to investigate the prediction of rock strata deformation and subsidence. Aiming to monitor the overburden deformation in a specific coal mine, an indoor physical model of similar material was built. By using DFOS technology, the deformation characteristic was investigated under continuous coal mining, and the machine learning method was introduced to build the deformation prediction model. By comparing the prediction value obtained by the prediction model with the physical model test measured value and finding that the coincidence degree of the two methods was as high as 97%, the investigation conclusions fully demonstrate the feasibility and accuracy of overburden deformation prediction based on the fusion technologies of DFOS and machine learning [49,50,51,52,53], which provide a new method for overburden failure law and “two-zone” (caving zone and fracture zone) height prediction. The prediction model modeling process is shown in Figure 26.

#### 3.3.4. Early Warning Layer

In the reduction and prevention of disasters caused by coal mining, perception is just a means, while effective early warning is the ultimate goal. For the early stage of investigation and treatment of coal mining disasters, the early identification of overburden movement caused by coal mining was primarily dependent on manual observation by professionals with expertise, supplemented by the ground drilling flushing fluid method, drilling water injection leak detection method, and geophysical detection method [54,55].

The differences in geology and coal seam occurrence conditions among coal-producing areas and the uneven experience of observers, especially in the identification of mining disasters in coal mines with complex geological conditions, high mining depth, and high mining height, often lead to misjudgment and misinterpretation, which makes the identification accuracy low for a long time. Therefore, for mining disasters caused by overburden movement and collapse, not only should attention be paid to the interpretation and characterization of the sensing data of overlying rock–soil bodies but the focus must also be on the early warning and response to mining disasters caused by the fracture of strata. A large number of indoor tests and field measurement studies have shown that the type of monitoring technology and the quantity of sensors are not positively correlated with the success rate of early warning of mining disasters and accidents. Therefore, in the process of monitoring and investigating mining-induced overburden deformation, it is necessary to design specific monitoring schemes and set specific evaluation indicators, and the formulation of warning standards must be combined with other relevant factors, including geological lithology, mining depth, mining height, and mining speed [56,57,58,59,60].

**Figure 27:**
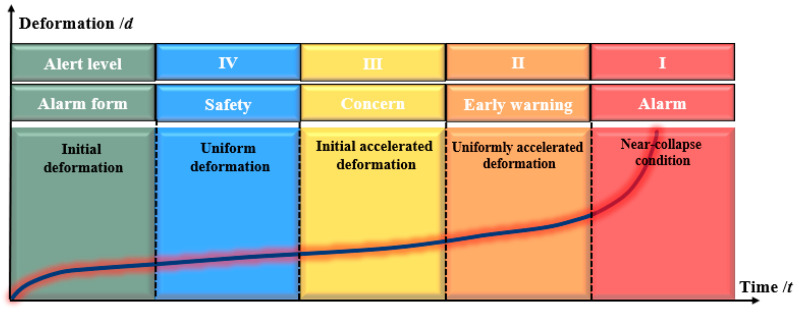
Early warning levels for overburden stability.

Due to the periodic and abrupt evolution laws of the movement and collapse of the overlying rock–soil body under the mining action, the monitoring and early warning of periodic movement and collapse can be achieved based on the conventional sensing data and the overburden failure theory. However, for the sudden deformation and failure characteristics of early warning, due to the sudden failure of the rock stratum, coupled with the fast speed and often accompanied by the overall shear of the rock block, the conventional sensing data miss detection, so it is difficult to directly identify the sudden change characteristic value through the distributed sensing data, and there is the problem of the threshold diversity of different types of overburden deformation monitoring and early warning indicators. Therefore, in terms of the monitoring, early warning, and emergency response of mining-induced overburden failure, the spatiotemporal continuous data of the whole process of overburden deformation and failure evolution can be obtained by the automatic frequency conversion monitoring technology based on intelligent perception, that is, a large sampling time interval is automatically maintained in the initial deformation and constant velocity deformation stage of overburden. Once the movement of the overburden enters the acceleration stage, the sampling time interval is automatically reduced, especially at the critical transition stage. The monitoring system will continuously collect various parameters to improve the accuracy of overlying strata disaster early warning and automatically send the critical crossing alarm so as to strive for more rescue time for emergency rescue and disposal of mining disasters and accidents and ensure that disaster losses are minimized. Cheng et al. [61], based on the evolutionary law of the deforming and collapsing of the overburden during coal mining under typical geologic conditions, and considering the formation lithology, mining height, and mining rate, determined the corresponding critical threshold by identifying the critical characteristics and divided the overburden stability into four levels: safety, concern, early warning, and alarm (Figure 27). In the future, by building a predictive model based on the fusion of multifield data, the real-time monitoring and reliability of the integrated safety guarantee system can be improved throughout the coal mining life cycle, turning passive early warning into active prevention and control.

#### 3.3.5. Decision Layer

The decision-making layer of the integrated system is the central center for controlling and managing the entire system. According to the results from the processing layer, combined with the warning level and corresponding threshold of the warning layer, it will integrate, analyze, process, and judge the information at each level and formulate scientific and reasonable decision-making plans on this basis. The basic process of the layer is as follows: (1) Secondary processing and analysis of data. The layer first receives data from various layers such as the processing layer and the early warning layer, including real-time data, historical data, and external data. After preprocessing steps like noise removal and format conversion, the data undergoes data mining and statistical analysis. (2) Decision and assessment. According to the data processing results and their corresponding warning levels, make a preliminary assessment of the existing conditions and threats facing the target. Develop a scientific decision-making plan based on problem-level analysis and assess the decision-making plan from aspects such as prediction of decision-making effectiveness, decision-making risks, and implementation difficulty. (3) Decision control and coordination. Through the overall planning of the system’s decision–making level, control, and collaboration among various functional layers are coordinated to ensure smooth operation and efficient collaboration between layers. Furthermore, based on the decision-making plan, system resources and technical support are allocated reasonably. (4) Assisted decision making and intelligent management [62,63]. By precisely selecting information-based decision support tools and methods such as decision trees and expert systems, the online management mechanism is established to transform the process of decision making from manual formulation to intelligent-assisted decision making. This enables the intelligent matching of optimal solutions and the creation of a problem database and solution library. This library can quickly provide decision-making solutions for subsequent similar problems, realizing the intelligent management of decision-making knowledge. (5) Decision report and decision feedback. Based on the final decision outcome mentioned above, corresponding decision report documents are automatically generated online, enabling decision-makers to download them anytime and anywhere. Additionally, the online evaluation function is utilized to collect the issues list encountered during decision execution, providing a valuable reference for the adjustment and optimization of the decision scheme.

**Figure 28:**
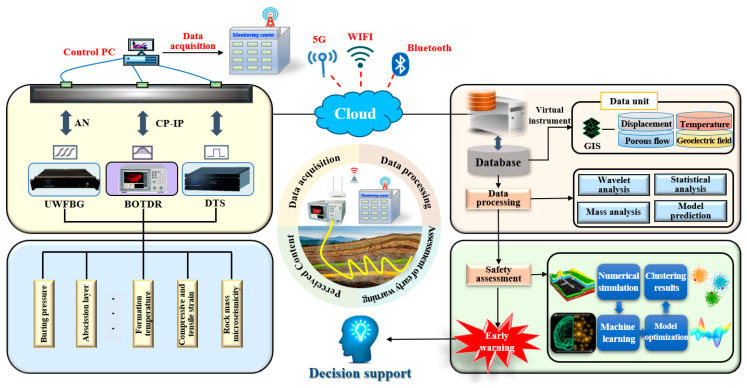
Integrated spatiotemporal continuous sensing system.

To sum up, establishing an integrated spatiotemporal continuous sensing system for overburden deformation that covers the entire coal seams mining cycle is of great significance for ensuring safe mining. This system integrates functions such as multisource data perception, instant transmission, efficient processing, real-time warning, and decision support. It can also send disaster risk warning information and emergency response measures in real time through the cloud. Furthermore, the system incorporates expandable functions that can potentially leverage machine learning-based multifield data fusion technology for mining-induced overburden rocks [64,65,66] in the future. This will address the issues of three-dimensional real-time visualization and numerical simulation of sensing data from the overlying rock–soil body under mining activity, as illustrated in Figure 28. Simultaneously, leveraging the massive periodic data accumulated by the system, research was conducted on machine learning and model optimization for the deformation modes of overburdening to achieve the full process prediction of the evolution and development potential of coal seam overburden deformation to ground subsidence under mining disturbance.

## 4. Conclusions

There are a large number of interface discontinuities in overburdened coal mining showing characteristics of nonlinear deformation. Consequently, it is of significance to proceed with research on the spatiotemporal continuous fiber optic neural perception system of overburden deformation and mining subsidence, obtain the spatiotemporal distributed data of the overlying strata in real-time and with accuracy, and clarify the whole process of force change in the overburden compression–tensile strain transition zone to ensure the safe production and the protection of the ground environment.

(1) Accurate and reliable acquisition of spatiotemporal continuous deformation information of the rock–soil body above coal seam under mining is the basis for the realization of safe mining monitoring and early warning. In this paper, based on the review of the development and evolution stages of the stope theory, the advantages and disadvantages of the in situ observation technology of mine rock and soil mass were compared and analyzed from five levels: survey, remote sensing, testing, exploration, and monitoring.
(2) The evolution characteristics and failure mechanism of the compression–tensile strain transition zone form three aspects: the typical mode of overburden deformation, the key controlling factors of deformation and failure in the overburden compression–tensile strain transition zone, and the stability assessment of overburden based on reliability theory, thereby realizing the accurate description of the development characteristics and distribution mode of overburden fractures.
(3) On the basis of the comparative analysis of the mainstream “3Ds” technology, the spatiotemporal continuous information perception technology for overburden deformation based on DFOS was introduced in detail, the judgment criteria of the coupling performance of sensing cable and rock–soil body for overburden deformation was investigated, and a proposal to realize the accurate assessment of coal mining disaster risk and the reliable prediction of ground subsidence potential by constructing an integrated safety guarantee system based on fiber optic neural perception network was presented.
(4) With the rapid development of AI, the monitoring technology of overburden deformation has gradually developed from the traditional periodic manual operation to real-time automatic monitoring, from the single underground overburden deformation monitoring to the integrated full-dimensional monitoring of “air–space–ground-interior”, and it continues to develop in the direction of full real-time, regional, and refined visualization and digital intelligence. In the future, various AI algorithms should be vigorously introduced to carry out machine learning on the long-term accumulated data of overburden deformation under different geological conditions and continuously revise the prediction model of the failure evolution process of overburden, as well as the development trend of mining subsidence, so as to realize the accurate prediction of the failure mode and catastrophic process of overlying rock strata under different geological conditions.

## Figures and Tables

**Table 1:** Comparison of in situ observation technology.

Method	Overview	Technology	Advantages	Limitations
Survey	Surveys are used for the observation of geotechnical surfaces. That is, the degree of deformation is judged by on-site observation of roof plate, settlement, and rock mass characteristic changes.	Surveyors can observe the rock–soil body with the naked eye or with the help of telescopes, optical microscopes and other tools.	The expenses are low, convenient, and fast, and the observation results are intuitive and easy to display.	Time and space are discontinuous, and it depend on the experience of surveyors.
Remote sensing	Mainly used for distribution monitoring of slopes in open pit mines and the deformation monitoring of underground mine mining subsidence. Through satellites, aviation aircraft, unmanned aerial vehicles (UAV) and other flight equipment, a large amount of surface position information is obtained.	High-precision and noncontact monitoring of the surface of rock and soil masses are carried out through the monitoring technology in various air-based (GNSS, BDS, GALILEO, GPS, etc.) and space-based (airship, UAV, etc.).	The wide measurement space, abundant data information, and high precision measurement.	It is challenging to obtain the deep changes in the rock–soil body, and a large amount of vegetation cover on the surface of the rock–soil body has a great impact on the measurement results.
Testing	Including in-situ testing and indoor geotechnical testing. The in-situ test is to determine the character of rock and soil while maintaining its natural structure, water content and stress state. The indoor geotechnical test is to understand its physical, chemical and mechanical character through various experiments.	Including seismic exploration, electromagnetic method, gravity method, magnetic method, sonic method, stress and deformation testing, etc.	The detection space is continuous, the accuracy is high, the destructiveness is low, the adaptability is strong, and the work efficiency is high.	The time is discontinuous, the environment is greatly affected, the operation is intricate, the expenses are high, and the test results are easily affected by humans.
Exploration	It mainly uses the anomalous behavior of rock and coal seams at physical locations to define the character of the rock–soil body. It is divided into drilling and geophysical prospecting, drilling is the use of boreholes to sample, observe, and test the deep rock–soil body; geophysical prospecting refers to detecting geological conditions and inverting the characteristics, changing process of the rock–soil body by studying and observing the changes in each physical field.	Including ground borehole observation method, ground borehole flushing fluid method, digital borehole TV method, downhole elevation angle borehole water injection and side leakage method, elastic wave logging method, inter-hole seismic wave (electrical) CT method, and resistivity method, etc.	The detection space is continuous, the equipment is light, the expenses are cheap, the efficiency is high, and the working space is wide.	The time is discontinuous, the observation data are limited and relatively discrete, the inversion results are multisolvable, and the spatial distribution rate is not high, so it is impossible to achieve high-precision detection.
Monitoring	Monitoring is making use of certain instruments, equipment and methods, to achieve real-time or regular monitoring, measurement, and analysis of the stress, deformation, seepage, temperature, cracks, and vibration, to obtain the engineering properties of the rock–soil body and its change law.	By using stress gauges, displacement gauges, water level gauges, temperature sensors, vibration sensors and other equipment to monitor various physical parameters within the rock–soil body.	Time continuous, distributed, long-distance, good durability, strong anti-interference, easy networking.	The data obtained are relatively small and discrete, and the monitoring space is discontinuous so that the spatiotemporal continuous characterization of the rock–soil body cannot be realized.

**Table 2:** Influencing factors of deformation and subsidence.

Type	Id	Influencing Factors
Geological factors	A_1_	Coal seam dip *α*/(°)
	A_2_	Loose layer thickness *H*_soil_/m
	A_3_	Bedrock thickness *H*_rock_/m
	A_4_	Comprehensive hardness of overburden *Q*
Mining factors	A_5_	Mining height *M*/m
	A_6_	Mining depth *H*_0_/m
	A_7_	Working face inclination length *D*_1_/m
	A_8_	The length of the face strike *D*_2_/m
	A_9_	Perturbation coefficient $n\;=\frac{\sqrt{D_{1}D_{2}}}{H_{0}}$
Additional factors	A_10_	Superficial engineering construction
	A_11_	Groundwater extraction

**Table 3:** Comparative analysis of various fiber optic sensing technologies (in the “Parameters”, D represents distance, S represents strain, T represents temperature, V represents vibration).

Classification	Parameters	Principles	Advantages and Disadvantages	Application Field
FBG	S, T	By fabricating Bragg grating structures in optical fibers and studying the changes in their reflection spectra, the temperature can be measured.	Anti-electromagnetic interference; Corrosion resistance; High sensitivity. High monitoring cost and need to eliminate the influence of strain (temperature).	① Ground subsidence, bridge and tunnel deformations; ② aircraft wing and ship structural deformation; ③ oil and gas pipeline leaks.
UWFBG	S, T, V	When incident light enters the fiber, once the UWFBG is subjected to an external physical field, the wavelength will change. By detecting the wavelength of the reflected light, the physical parameters can be determined.	Multiple data; high accuracy; wide applicability; corrosion resistance; the ability to achieve dense distributed dynamic measurement. Complex production process; high cost; high requirements of the demodulation performance.	① Safety monitoring in the development, storage, and transportation of various energy; ② monitoring of various geological disasters; ③ safety monitoring of various structural engineering.
OTDR	D	By transmitting an optical pulse into the fiber, the backscattered light reflected from various locations is received, the distance and loss in the fiber are measured.	Single-ended measurement; fast measurement; precise positioning. High operator requirements; harsh testing environment; low spatial resolution.	① Fault diagnosis of communication cables; ② quality inspection of optical cable production; ③ optical imaging and biomedicine.
OFDR	S, T	By measuring the frequency, the optical intensity at each position can be obtained, enabling the identification of splices, bends, and breaks. According to the frequency shift, the strain (temperature) can be measured.	High measurement accuracy; strong anti-electromagnetic interference; high spatial resolution. Short testing distance; high requirements of the testing environment.	① Exploration of oil and gas resources, monitoring of structural health; ② medical minimally invasive interventional surgery.
ROTDR	T	Due to anti-Stokes, light is sensitive to temperature. By measuring the intensity ratio, the temperature distribution can be obtained.	Corrosion resistance; high temperature resistance; fast speed; Long distance. High cost; limited measurement accuracy (special application scenarios).	① Monitoring of coal mine temperature; ② monitoring of landslide temperature field; ③ monitoring of concrete pouring.
DAS	V	Interference occurs between the incident light and the backscattered light. When there are changes in sound or vibration, it will cause a linear variation. Thus, the amount of change at that point can be determined.	Strong anti-interference ability; flexible deployment; high concealment; long-distance distributed measurement. High cost; limited monitoring accuracy (complex environments).	① Monitoring of oil and gas pipeline leaks; ② perimeter security and environmental monitoring; ③ monitoring of geological exploration and building health.
BOTDR	S, T	The incident light interplays with the acoustic phonons, which produce backscattered light. When there is a change in temperature or strain, the variation can be calculated according to frequency shift.	Single-ended measurement; wide monitoring range; strong anti-electromagnetic interference; excellent environmental adaptability. Limited spatial resolution makes it difficult to achieve precise measurements.	① Monitoring of energy exploration; ② monitoring of submarine cable fault location; ③ monitoring of geological disaster prevention and control.
BOTDA	S, T	Pump light and continuous light excitation waves are injected into both ends of the fiber, producing stimulated Brillouin scattering, the variation is obtained based on the relationship between frequency shift and strain (temperature).	The accuracy and spatial resolution of Brillouin Optical Fiber Time Domain Analysis (BOTDA) are significantly higher than BOTDR. Need to construct a testing circuit; poor environmental adaptability; high deployment costs for the monitoring system.	① Safety monitoring of pipelines, bridges and tunnels; ② Monitoring of groundwater level changes, oil and gas pipeline leaks; ③ indoor physical model tests for various geological disasters.
BOFDA	S, T	Pump light and Stokes light are injected into both ends of the fiber; the variation is compared with the initial phase and amplitude to calculate the correspondence between the position and the frequency shift.	The measurement accuracy and spatial resolution are slightly higher than BOTDA. A testing circuit needs to be constructed, with slightly lower repeatability and stability than BOTDA.	① Pile foundation monitoring; ② maintenance of power systems; ③ construction and operation of tunnels, and structural health monitoring.

## Data Availability

The data are available from the corresponding author on reasonable request.
